# Gut microbiota-derived short-chain fatty acids regulate gastrointestinal tumor immunity: a novel therapeutic strategy?

**DOI:** 10.3389/fimmu.2023.1158200

**Published:** 2023-04-14

**Authors:** Yue Dong, Kexin Zhang, Jingge Wei, Yiyun Ding, Xin Wang, Huiqin Hou, Jingyi Wu, Tianyu Liu, Bangmao Wang, Hailong Cao

**Affiliations:** Tianjin Key Laboratory of Digestive Diseases, Tianjin Institute of Digestive Diseases, Department of Gastroenterology and Hepatology, Tianjin Medical University General Hospital, Tianjin, China

**Keywords:** short-chain fatty acids, gastrointestinal tumors, tumor immunity, probiotics, fecal microbiota transplantation, immunotherapy

## Abstract

Tumor immune microenvironment (TIME), a tumor-derived immune component, is proven to be closely related to the development, metastasis, and recurrence of tumors. Gut microbiota and its fermented-metabolites short-chain fatty acids (SCFAs) play a critical role in maintaining the immune homeostasis of gastrointestinal tumors. Consisting mainly of acetate, propionate, and butyrate, SCFAs can interact with G protein-coupled receptors 43 of T helper 1 cell or restrain histone deacetylases (HDACs) of cytotoxic T lymphocytes to exert immunotherapy effects. Studies have shed light on SCFAs can mediate the differentiation and function of regulatory T cells, as well as cytokine production in TIME. Additionally, SCFAs can alter epigenetic modification of CD8^+^ T cells by inhibiting HDACs to participate in the immune response process. In gastrointestinal tumors, the abundance of SCFAs and their producing bacteria is significantly reduced. Direct supplementation of dietary fiber and probiotics, or fecal microbiota transplantation to change the structure of gut microbiota can both increase the level of SCFAs and inhibit tumor development. The mechanism by which SCFAs modulate the progression of gastrointestinal tumors has been elucidated in this review, aiming to provide prospects for the development of novel immunotherapeutic strategies.

## 1 Introduction

Accumulating evidence indicates that tumors have become the leading cause of death. GLOBOCAN counted the global cancer situation and found that the burden of cancer is increasing rapidly worldwide (1). It is universally acknowledged that gut microbiota is interrelated to the occurrence and development of tumors, especially gastrointestinal tumors. Gut microbiota can influence the gut microenvironment by modulating specific bioactive metabolites of bacteria (2). Short-chain fatty acids (SCFAs), as typical products of soluble fiber fermented by gut bacteria, exert critical functions in gut homeostasis. SCFAs can regulate energy metabolism, strengthen the intestinal barrier and exert anti-inflammatory properties. As key regulators of immune function, SCFAs can regulate T cells, B cells, macrophages, and other immune cells (3). Because of the combination of G protein-coupled receptors (GPCRs) or suppression of histone deacetylases (HDACs), SCFAs can affect the signal transduction pathway of immune response and modulate the release of immune-related inflammatory mediators, thus regulating the tumor immune microenvironment (TIME) (4).

TIME, consisting of tumor-infiltrating immune cells, tumor-associated other cells, tumor cells, and extracellular matrix, attracts more and more attention (5). Especially, immune components serve a critical regulative function. The treatment scheme based on the immune system, such as immune checkpoint inhibitors (ICIs), has been applied in the clinic (6, 7). In recent years, the effect of SCFAs on the TIME has been widely studied. Depending on HDAC inhibitor activity, SCFAs can directly affect T cell differentiation and function. In colorectal cancer (CRC) and pancreatic cancer experimental animal models, it has been demonstrated that butyrate could enhance the anti-tumor effect of CD8^+^ T cells (8, 9). Targeting tumor immunity, supplementation of microbiota-derived SCFAs has become a new way to diagnose, treat, and prevent tumors. Future work can focus on probiotics and fecal microbiota transplantation (FMT), improve the level of SCFAs, regulate gastrointestinal microecology, and activate effective anticancer effects.

## 2 SCFAs, immune cells and immune microenvironment

Recently, more and more attention is given to the influence of microorganisms and microbial metabolites on the host. SCFAs, consisting of less than 6 carbon numbers, are typical metabolites produced by symbiotic bacteria through fermentation of dietary fiber in the gastrointestinal tract and have been widely studied. The production of SCFAs is a complex process, which is carried out in the colon by a variety of bacteria (**Table 1**). Among all SCFAs, acetate, propionate, and butyrate are the most representative.

**Table 1:** Short chain fatty acids and associated-microbiota in tumor immune microenvironment. (Bacteria data sources: gutMGene).

SCFAs	SCFA-producing bacteria	Effects and mechanisms
Acetate	*Akkermansia muciniphila; Bacteria Latreille; Bacteroidetes; Bacteroides thetaiotaomicron; Barnesiella intestinihominis; Bifidobacterium dentium; Bifidobacterium longum; Blautia faecis; Christensenella minuta; Clostridium asparagiforme; Clostridium pasteurianum; Clostridium scindens; Clostridium* sp.*; Collinsella tanakaei; Enterococcus casseliflavus; Eubacterium ramulus; Eubacterium limosum; Lawsonibacter asaccharolyticus; Ruminococcus champanellensis; Succinatimonas hippie; Succinivibrio dextrinosolvens*	Enhanced production of ROS and expression of MCT1 through oxidative stress to induce GC cell apoptosis (10);
		Elevated the effect of PDT by inducing cancer-specific HCP1 expression *via* ROS production (11);
		Promoted lysosomal membrane permeabilization and cathepsin D release to induce CRC cell apoptosis (12);
		Reduced proliferation and glycolysis and increased both oxygen consumption and ROS levels in CRC cells (13);
		Reduced the expression of PVR/CD155 by deactivating the PI3K/AKT pathway to enhance effector responses of CD8^+^ T cells in CRC (8);
		Reduced IL-17A-producing ILC3s infiltration and enhanced the antitumor immunity of immune checkpoint inhibitors in HCC (14).
Propionate	*Acidipropionibacterium acidipropionici; Akkermansia muciniphila; Bacteroides; Bacteroides thetaiotaomicron; Dialister succinatiphilus; Eubacterium limosum; Haemophilus parainfluenzae; Parasutterella excrementihominis; Phascolarctobacterium succinatutens; Propionibacterium avidum; Roseburia inulinivorans; Ruminococcus bromii; Veillonella; Veillonella ratti*	Raised barrier proteins FLG and DSG1 expression to restore interleukin 13-compromised esophageal epithelial barrier function, same as butyrate (15);
		Induced apoptosis and necrosis in Kato III cells and arrested cells in the G2-M phase by increasing OPP activity and decreasing GSH availability, coincided with butyrate (16);
		Upregulated surface expression of the immune stimulatory NKG2D ligands MICA/B and caused metabolic stress in CRC (17);
		Downregulated histone arginine methyltransferase PRMT1 levels to induce CRC cell apoptosis (18);
		Promoted the proteasomal degradation of EHMT2 through HECTD2 upregulation to suppress CRC growth (19);
Butyrate	*Atopobium parvulum; Butyricimonas synergistica; Butyricimonas virosa; Christensenella minuta* *Clostridium; Clostridium butyricum; Clostridium pasteurianum; Clostridium tyrobutyricum; Enterococcus durans; Eubacterium hallii; Eubacterium limosum; Eubacterium ramulus; Eubacterium rectale; Faecalibacterium prausnitzii; Firmicutes; Fusobacteriia; Lawsonibacter asaccharolyticus; Prevotella copri; Roseburia inulinivorans; Roseburia* sp.*; Salmonella enterica*	Activated the mitochondrial apoptosis-related pathway to enhance GC cell apoptosis by combining with cisplatin or alone (20);
		Regulated miRNAs and related oncogenic pathways to inhibit the proliferation and migration in KATO III cells (21);
		Induced the expression of tumor suppressor genes Per1 and Per2 in human GC cells by inhibiting HDACs (22);
		Induced the apoptosis and autophagy and inhibited of CRC cell proliferation, invasion, and metastasis through the following mechanisms: Activating LKB1/AMPK/ACC axis (23); Inducing cell cycle arrest at the G2 phase with a drop in S-phase fraction (24); Enhancing miR-200c expression-mediated downregulation of BMI-1and reversing EMT (25, 26); Regulating KEAP1/NRF2 signaling and driving metabolic rewiring, CpG methylomic, and transcriptomic reprogramming (27); Activating PKM2 *via* promoting its dephosphorylation and tetramerization and reprogramming the metabolism (28); Blocking the activation of AKT1 and ERK1/2 by inhibiting HDAC3 (29); Decreasing the expression of LHX1 gene by inhibiting HDAC8 (30, 31); Enhancing the inhibition of SIRT1 silencing on cell proliferation and activity of mTOR/S6K1 (32); Decreasing Trx-1 transcription (33); Degrading β-catenin (34);
		Upregulated mucosal repair factors and stimulated protective responses against oxidation and inflammation by combing GPR109A to alleviate pathological damage to gastric mucosa (35);
		ECM-integrin/PI3K axis may mediated phenotypic changes in the NaB-treated CRC organoid (36);
		Decreased CRC burden by decreasing IL-6 receptor gp130 and blocking IL-6/JAK2/STAT3 axis activation (37);
		Increased production of ATP by oxidative phosphorylation, enhanced mitochondrial spare respiratory capacity and caused rearrangement of the cellular phosphotransfer networks to modulate metabolic plasticity in differentiation of CRC cells (38);
		Reduced the abundance of membrane GLUT1 and cytoplasmic G6PD regulated by the GPR109a-AKT signaling pathway to inhibit glucose transport and glycolysis of CRC cells (39);
		Modulated SIRT-1/Ac-p53 regulatory axis to inhibit HBV replication and HCC cell proliferation (40);
		Downregulated signaling pathways altered by HBx and increased expression of a tumor suppressor called disabled homolog 2 to delay the pathogenesis of CLD and development of HCC (41);
		Decreased the expression of both sorafenib-targeted miR-7641 and miR-199 in HCC (42);
		Modulated the over-expression level of prostaglandin EP4 receptors and excessive induction of cyclooxygenase-2 in CRC (43);
		Inhibited the expression of HK2 to downregulate aerobic glycolysis and the proliferation of HCC cells and induced their apoptosis *via* the c-myc pathway (44);
		Augmented the previously described effects of HDAC6 inhibitors on CCA cell proliferation and migration (45);
		Reversed the CD11b+ cell-mediated T cell suppression to delay the progression of pancreatic ductal adenocarcinoma (46);
		Increased p16INK4a, p14ARF, and p15INK4b and decreased class I and II HDACs to inhibit cell growth and induce apoptosis in pancreatic cancer AsPC-1 and colon cancer HCT-116 cell lines (47).
Pentanoate	*Clostridioides difficile; Megasphaera massiliensis (* 48)	Increased the function of mTOR as a central cellular metabolic sensor and inhibited class I HDAC activity to enhance the anti-tumor activity of CTLs and CAR T cells (9), same as butyrate;
		Slowed proliferation, promoted apoptosis, improved intestinal integrity and microbiota composition, and affected cancer stromatogenesis, serum metabolome and lipidome in pancreatic cancer with or without gemcitabine (49).

ACC, acetyl coenzyme A carboxylase; Ac-p53, Acetylated p53; AKT, protein kinase B; AMPK, AMP-activated protein kinase; BMI-1, B cell-specific MLV integration site-1; CAR, chimeric antigen receptor; CCA, cholangiocarcinoma; CLD, chronic liver disease; CRC, colorectal cancer; CTLs, cytotoxic T lymphocytes; DSG1, Desmoglein 1; ECM, extracellular matrix; EHMT2, euchromatic histone-lysine N-methyltransferase 2; EMT, epithelial-mesenchymal transition; ERK1/2, extracellular signal-regulated kinase 1/2; FLG, filaggrin; G6PD, glucose-6-phosphate dehydrogenase; GC, gastric cancer; GLUT1, glucose transporter type 1; GSH, glutathione; HBV, hepatitis B virus; HCC, hepatocellular carcinoma; HDACs, histone deacetylases; HECTD2, HECT domain E3 ubiquitin protein ligase 2; IL, interleukin; ILC3s, group 3 innate lymphoid cells; JAK2, Janus Kinase 2; KEAP1, kelch-like ECH-associated protein 1; LHX1, LIM homeobox 1; LKB1, liver kinase; MCT1, monocarboxylic transporter 1; MICA/B, MHC class I polypeptide-related sequence A/B; mTOR, mechanistic target of rapamycin; NK, natural killer; NKG2D, type II integral membrane protein; NRF2, nuclear Factor erythroid 2-Related Factor 2; OPP, Oxidative pentose pathway; PDT, photodynamic therapy; PI3K, phosphatidylinositol-4,5-bisphosphate 3-kinase; PKM2, pyruvate kinase M2; PRMT1, protein arginine methyltransferase 1; PVR, poliovirus receptor; ROS, reactive oxygen species; S6K1, ribosomal protein S6 kinase beta-1; SIRT1, silencing information regulator 2 related enzyme 1; STAT3, signal transducer and activator of transcription 3.

**Figure 1:**
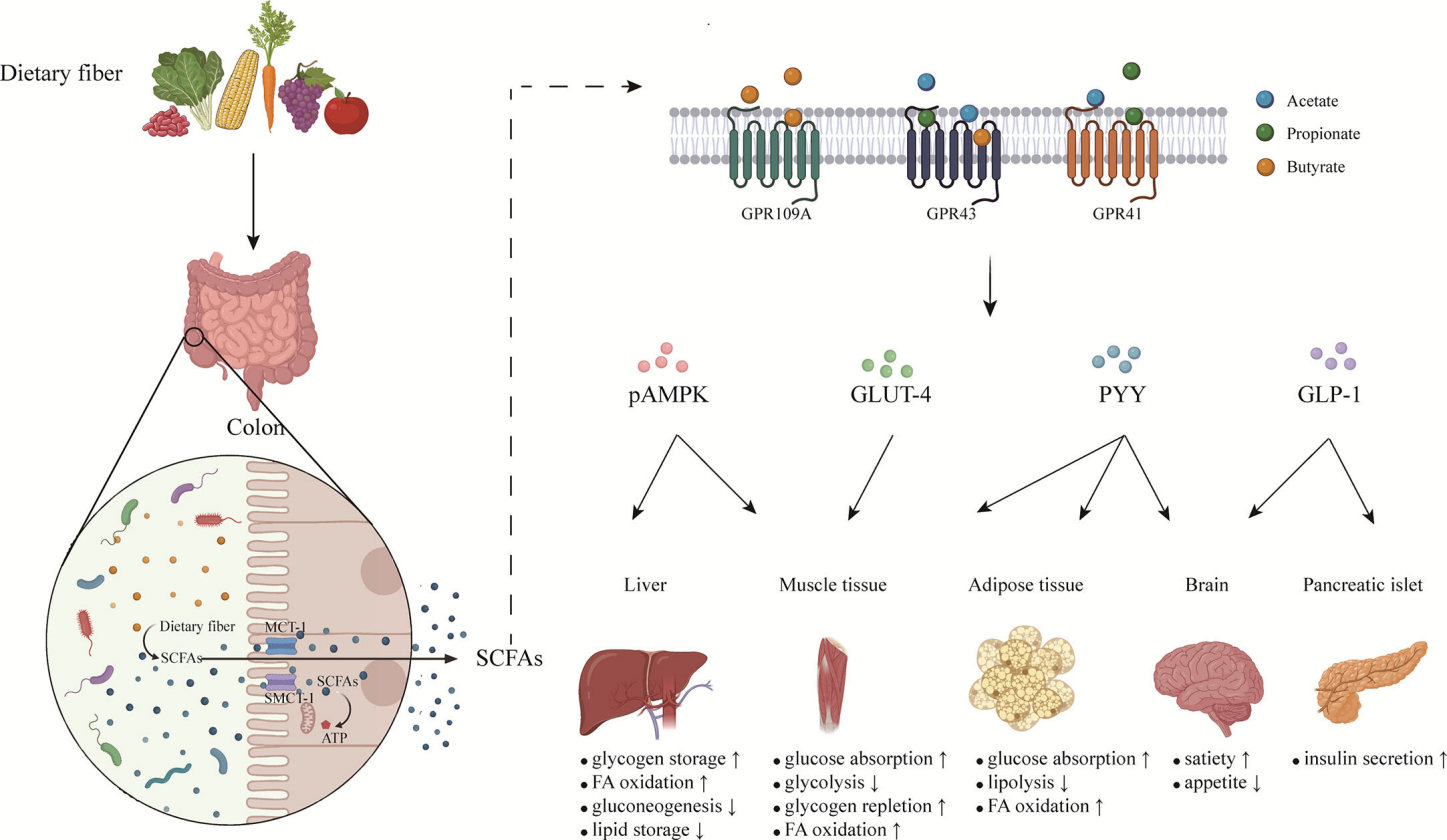
The effects of short chain fatty acids on metabolic homeostasis. SCFAs are produced by gut microbiota fermenting soluble dietary fiber in colon. Most SCFAs are absorbed by colonocytes *via* MCT-1 and SMCT-1, while small part of SCFAs pass directly through the intestinal barrier by passive diffusion. SCFAs transported to various organs of the whole body exert different functions mainly by binding with GPCRs on cells. By coordinating various organs and systems, SCFAs regulate energy balance and maintain metabolic homeostasis. SCFAs, short chain fatty acids; MCT-1, monocarboxylate transporter 1; SMCT-1, sodium-coupled monocarboxylate transporter 1; GLP-1, glucagon-like peptide-1; PYY, peptide tyrosine tyrosine; pAMPK, phosphorylated adenosine monophosphate activated protein kinase; GLUT-4, glucose transporter-4; FA, fatty acids.

When the SCFAs are produced, the first function is to serve as substrates to provide energy. Most SCFAs are absorbed by colonocytes *via* two transporters: the monocarboxylate transporter 1 (MCT-1) and the sodium-coupled monocarboxylate transporter 1 (SMCT-1) (50). SCFAs are transferred in an H+-dependent, electroneutral manner by MCT-1, whereas SCFA anions are transported *via* the electrogenic SMCT-1. In addition to powering the colonocytes, SCFAs are transported to various tissues and organs of the whole body through blood transportation and then regulate biological responses in two main mechanisms (51). On the one hand, SCFAs decrease the activity or expression of HDACs, contributing to increased histone acetylation. It is reported that abnormal activation of HDACs exists in several types of cancer (51). On the other hand, SCFAs combine with the GPCRs, mainly for GPR41 (renamed free fatty acid receptor (FFAR)3), GPR43 (renamed FFAR2), and GPR109A, to exert corresponding signal cascade effects (**Figure 1**). Some studies have shown that abnormal expression or activity of GPCRs is involved in various tumor progression. Previous studies have summarized the role of SCFAs in regulating energy metabolism, protecting gut integrity, and ameliorating the inflammatory response (52), but the effects of SCFAs on the immune system have not attracted enough attention.

**Figure 2:**
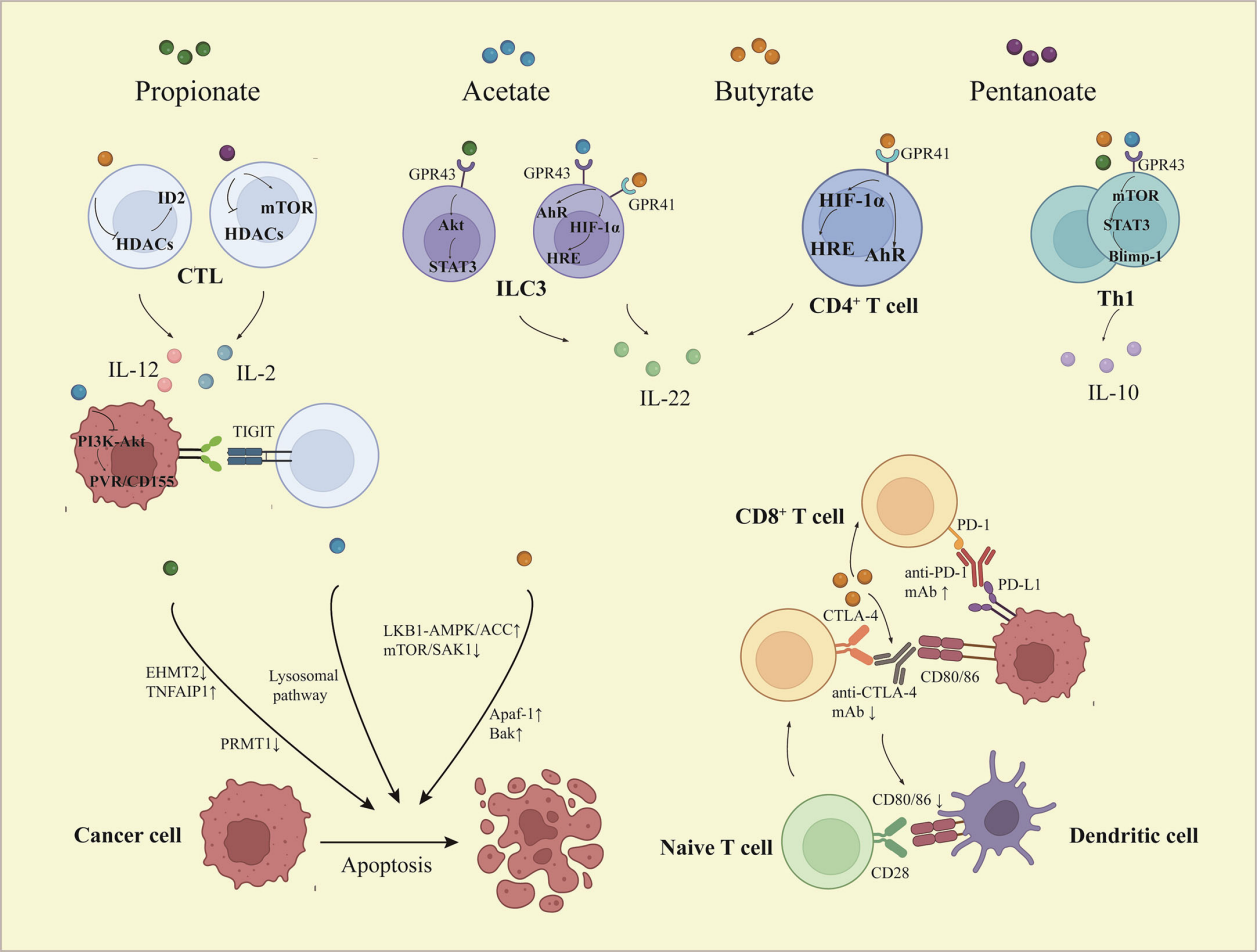
The effects of short chain fatty acids on tumor immune microenvironment. Depending on activation of STAT3 and mTOR, SCFAs promote Th1 cell expression of Blimp-1, which increases IL-10 expression. Butyrate and pentanoate promote the expression of IL-12 and IL-2 respectively in CD8+ T cells *via* HDACs inhibitory activity. Acetate decreases the expression of PVR/CD155 through inactivation of PI3K/AKT pathway, thus enhancing the response of CD8+ T cells. SCFAs induce IL-22 production in CD4+ T cells and ILCs, which is mediated by HIF-1α and AhR or involved by Stat3 and mTOR. Butyrate facilitates the anti-PD-1 mAb efficacy *via* regulating the T cell infiltration, while butyrate reduces efficacy of CTLA-4 blockade and limits DC maturation. Furthermore, SCFAs induce apoptosis of cancer cells through multiple ways. STAT3, signal transducer and activator of transcription 3; mTOR, mammalian target of rapamycin; Blimp-1, B lymphocyte-induced maturation protein 1; PI3K, phosphoinositide 3-kinase; AKT, the protein kinase B; PVR, poliovirus receptor; HRE, hypoxia response element; AhR, aryl hydrocarbon receptor; HIF-1α, hypoxia inducible factor-1α; IL, interleukin; Th, T helper; CTL, cytotoxic T lymphocyte; ILC, innate lymphocyte cell.

T cells are vital cells in TIME, especially decreased infiltration or dysfunction of T cells leads to poor clinical results in many cancer treatments (53). Since naive T cells expressed without GPR41 and GPR43 at functional levels, SCFAs could directly affect the differentiation of naive T cells relying on HDACs inhibitor activity. In this way, SCFAs promoted immature CD4^+^ T cells to differentiate into different regulatory and effector T cells, this was up to different polarization conditions which refer to cytokine phenotype and immunological milieu. SCFAs induced the activation of mTOR-S6K and STAT3 involved in the production of cytokines necessary for T cell differentiation. Whatever the polarization conditions, SCFAs promoted interleukin (IL)-10 expression, but could only facilitate the production of IL-17 or interferon-γ (IFN-γ) under specific environments (54). Interestingly, the secretion of IL-10 in differentiated T cells, such as T helper (Th)1 cell, depended on interaction with GPR43. In addition, SCFAs upregulated the expression of Blimp-1, which was associated with IL-10 production in Th1 cells and T cell function maintenance (**Figure 2**) (55). In high-fat diet-treated mice, the levels of IL-17 and IFN-γ were increased, while transforming growth factor-β (TGF-β) and IL-10 decreased. That meant higher differentiation of T cells toward Th17 and Th1 cells and lesser to T regulatory (Treg) cells. This situation could be reversed by SCFAs (56).

The discovery and investigation of IL-22 in CD4^+^ T cells and innate lymphoid cells could be modulated by SCFAs. Butyrate increased transcription factor HIF-1α and AhR expression through binding to GPR41, which was accompanied by STAT3 and mTOR activation. Meanwhile, butyrate-promoted HIF-1α combination with the HRE of the IL-22 promoter involved histone acetylation (**Figure 2**) (57). The process of CD4^+^ T cell differentiation is also subject to epigenetic regulation. Different CD4^+^ T cell subsets have specific transcription factors, such as T-bet for Th1 cells, RORγT for Th17 cells, and FOXP3 for Treg cells. Butyrate increased the expression of RORγT in differentiated Th17 cells by acetylating H4K16 but had no response to naive CD4^+^ T cells under the Th17 polarization condition (58).

Low butyrate concentration promoted the differentiation of CD4^+^ T cells to Foxp3^+^ Treg cells in a TGF-β1-dependent manner, while high butyrate concentration induced the T-bet expression and IFN-γ release no matter what conditions and subpopulations (59). This seemed to break our traditional understanding of the impact of SCFAs on mucosal immunity. More than that, exposure to high concentrations of SCFAs, especially butyrate, suppressed the proliferation and activation of CD4^+^ T cells and other CD4^+^ T cell subsets in the intestinal mucosa. This correlated strongly with histone acetylation and GPR43 activation (60).

Butyrate is involved in anti-tumor immunity by promoting CD8^+^ T cell effects. The promotional effect of cytotoxic T lymphocytes (CTLs) mediated anti-tumor responses treated with butyrate was dependent on a transcriptional regulator called ID2, whose level was much higher in CD8^+^ T cells in TIME. By inhibiting the activity of HDACs, butyrate induced ID2 expression and started the ID2-IL-12 signal pathway to improve the chemotherapy effect of oxaliplatin (61). Pentanoate enhanced the expression of IL-2, tumor necrosis factor-α (TNF-α), IFN-γ, and other effector molecules in CTLs *via* inhibition of HDAC activity (9). PVR/CD155 regulators are overexpressed in malignant tumors and bind to a T-cell immunoreceptor with Ig and ITIM domains to mediate immune escape. Acetate suppressed the level of PVR/CD155 by inhibiting PI3K/AKT pathway to enhance the anti-tumor ability of CD8^+^ T cells (**Figure 2**) (8). Activated γδ T cells are the main endogenous source of IL-17. Propionate repressed the secretion of IL-17, IL-22, and other cytokines in human γδ T cells by inhibiting HDACs, thereby preventing cancer progression (62).

## 3 SCFAs, gastrointestinal tumors, and tumor immunity

The abundance of SCFA and SCFA-producing bacteria in gastrointestinal tumors is significantly lower, and supplementation of SCFAs can inhibit the development of gastrointestinal tumors through a variety of mechanisms (**Table 1**).

### 3.1 Esophageal cancer

Research has shown that the destruction of mucosal barrier function is a high-risk factor for esophageal cancer. Propionate and butyrate treatment reversed the damaged esophageal epithelial barrier driven by IL-13. After treatment, the expression of barrier proteins FLG and DSG1 were significantly increased. HDAC antagonist occurred a similar effect, while GPCRs agonists did not, which means this function of SCFAs may be achieved by inhibiting HDAC activity (15). The alterations of gut microbiota composition in patients with esophageal squamous cell carcinoma could be observed, butyrate-producing bacteria decreased, while pro-inflammatory and carcinogenic bacteria increased (63). Additionally, clinical research showed that the concentrations of acetate and propionate in patients with complications after esophagectomy were significantly reduced, and preoperative supplementation of SCFAs may prevent infection and other complications (64).

### 3.2 Gastric cancer

Lower concentrations of propionate and butyrate were detected in the plasma of patients with gastrointestinal metaplasia or gastric cancer (65). It would appear that SCFAs could be used to evaluate the progression of gastric cancer. Besides, butyrate inhibited the proliferation and migration of the KATO III in a dose-dependent manner, which was associated with its effect on regulating microRNA (miRNA) regulatory networks (66). Taking *Clostridium butyricum* (*C. butyricum*) after gastrectomy could increase the concentrations of SCFAs, enhance immunity, reduce inflammation, and prevent postoperative complications (67). Vivo experiment demonstrated that acetate induced the apoptosis of gastric cancer cells, and subsequently, oxidative stress played an essential role has been proved *in vitro*. A large amount of acetate intake increased ROS production and MCT1 expression in gastric cancer cells (10). The over-expression of ROS upregulated HCP1, both of which resulted in the increase of porphyrin intake by gastric cancer cells. As a photosensitizer of photodynamic therapy, excessive absorption of porphyrin caused by acetate enhanced the efficacy (11). Epigenetic modifications may be one of the mechanisms that regulate the development of gastric cancer. Serving as an HDAC inhibitor, butyrate modified tumor suppressor genes Per1 and Per2 and induced their expression in the KATO III and NCI-N87 (22). The traditional remedy used in combination with SCFAs appeared to have better reactions and less toxicity. In the nude mouse xenograft tumor model, butyrate-cisplatin treatment suppressed the growth, migration, and invasion of gastric cancer cells and accelerated apoptosis relying on the mitochondrial apoptotic pathway (20). In addition, butyrate alone can induce apoptosis of gastric cancer cells by mitochondrial pathway, which has been confirmed in human cell lines BGC-823 and SGC-7901 (68).

### 3.3 Colorectal cancer

Previous studies revealed the concentrations of acetate, propionate, and butyrate in the population at high risk of CRC were significantly reduced, and the incidence of CRC was higher in individuals with lower SCFA levels than in healthy individuals (69). All kinds of SCFAs exhibited anti-cancer behaviors. Compared with treating alone, the compounds had a superposition effect (70).

#### 3.3.1 Acetate

Acetate can enhance apoptosis and reduce proliferation in cancer cells and has been confirmed in different CRC cell lines (71), so it has become a key factor in the treatment of CRC, but the concrete mechanism remains unclear. In CRC patients, SCFAs were reduced and acetate metabolism was converted to acetyl-CoA (72). A past study showed that the acetate-mediated apoptosis was dependent on the lysosomal pathway triggered by partial lysosomal membrane permeabilization. However, the subsequent release of cathepsin D in the lysosome-dependent selective death pathway lowered the sensitivity of acetate. From these, cathepsin D inhibitors may be considered a good option combined with acetate (12). Appropriate regulation of mitochondrial function is essential for proliferation impeded by exogenous acetate in normoxic conditions. Specifically, acetate suppressed glycolysis and triggered ROS generation. However, increased proliferation of cancer cells with acetate occurred in the absence of oxygen, which relied on the up-regulation of ACSS2 and activation of HIF-2 (13). At the same time, acetate promoted the growth of HCT-116- and HT-29-derived tumors by activating the ACSS2/HIF-2 signaling pathway in the presence of glucose deficiency (73). At physiological concentrations, acetate promoted the growth of COLO 205 cells through activating AcK and enhancing oxidative phosphorylation and accelerated the proliferation of HCT-116 cells by elevating glycolysis. Nevertheless, butyrate or propionate drove apoptosis of two cell lines *via* the mitochondrial pathway at physiological doses (74). Thus, the anticancer effect of acetate can be altered in response to changes in environment and concentration. Moreover, acetate, as a regulator for immune checkpoint ligand PVR/CD155 driven by PI3K/AKT signaling, can enhance functional responses of CD8^+^ T cells in TIME and promote the production of IFN-γ, which is expected to become a related drug to promote tumor immunity. Based on the above discussion, the efficacy of ICIs may benefit from acetate (8).

#### 3.3.2 Propionate

The content of propionate in colorectal cancer tissue decreased, and the addition of propionate to the SW480 cell line significantly inhibited growth (75). SCFAs regulated immune stimulatory and inhibitory ligands and were involved in the immune cell-mediated killing. The upregulation of NKG2D ligands MICA/B induced by propionate depends on neither inhibition of HDACs nor a combination of GPR41/GPR43 receptors, but mitochondrial activity, while butyrate is decided by its HDACs inhibitor activity. This effect of propionate was closely related to the PEPCK-M enzyme and mTORC2/PDK1/AKT pathway which mediate tumor suppressor protein p21 expression (17). In addition to immune immunoregulation, epigenetic modulation is a promising target for SCFAs to play anti-cancer roles. Propionate resulted in down-regulated expression of PRMT1 by preventing p70 S6 kinase phosphorylation, leading to selective death of CRC cells (18). Furthermore, propionate induced the HECTD2 upregulation resulting in the degradation of EHMT2, thus promoting the expression of downstream TNFAIP1 and ultimately apoptosis (19). Epigenetic modification is non-negligible in propionate-mediated anticancer treatments. However, the latest mendelian randomization analysis found that there was no strong evidence to prove the correlation between the concentration of propionate in feces and the risk of colorectal cancer (76). It may be necessary to comprehensively detect SCFAs and their producing bacteria.

#### 3.3.3 Butyrate

It is universally acknowledged that butyrate inhibited the proliferation of CRC cells but nourished normal colon cell growth. CRC cells preferred glucose as substrate rather than butyrate, which was described as the butyrate paradox (77). That is, glycolysis replaced oxidative phosphorylation. Compared with other SCFAs, butyrate has a stronger inhibitory effect on CRC cell lines (78). On the one hand, butyrate can inhibit pro-inflammatory mediators TNF-α, IL-1β, IL-6, and IL-8, and up-regulate anti-inflammatory factor IL-10; on the other hand, butyrate can promote anti-tumor immunity by promoting CD8+T cells to play a role. In addition, butyrate maintains the integrity of the intestinal barrier by promoting epithelial cell proliferation, increasing the mucus layer, and improving tight junctions (79). In HCT116 cells, butyrate-mediated apoptosis was inseparable from p300-Wnt signaling (80). Among them, colon cancer cells were more sensitive to butyrate under the oncogenic Wnt signaling gene expression mode than the receptor-mediated Wnt signaling gene expression mode (81). The structure and stability of gut microbiota were significantly altered in CRC and intimately associated with its progression (82). Butyrate administration improved microecological disorders reflected in the decreased pathogens and the ratio of *Firmicutes* to *Bacteroidetes* and increased abundance of probiotics (83–87). In contrast to acetate and propionate, butyrate has a stronger effect on the regulatory networks which are essential to the cell cycle in CRC. Butyrate regulated the expression of cancer-related miRNA, of which miR-139 and miR-542 were well-known representatives. Specifically, they were conducted as collaborative objects of butyrate to modulate EIF4G2 and BIRC5 genes in the cell cycle (88). Butyrate regulated the c-Myc/p21 pathway to induce cell cycle arrest in the G2 phase, with the inclusion of 27 apoptosis-related genes (24). Moreover, butyrate triggered a cell cycle block at the G1 phase requiring a complicated lncRNA-miRNA-mRNA regulatory network (89). Butyrate supplementation reversed the overexpression of CSE1L and appeared to show synergy with p53, eventually arresting cancer cells at the G1 and G2/M phases (90). Meanwhile, cell cycle arrest in the G2/M phase occurred in response to butyrate-induced p21 and γ-H2AX increase, along with cyclin B1 decrease. Not only that but butyrate had an inhibitory effect on cancer cell migration by upregulating miR-200c and suppressing its direct target BMI-1 (25). BMI-1 was an essential regulator that induced epithelial-mesenchymal transition (EMT) dependent on AKT/GSK-3β/snail pathway to drive cancer metastasis, butyrate prevented the effect of BMI-1 (26). Treatment of butyrate decreased Trx-1 expression in CRC cells instead of normal colonocytes. Studies have proven that Trx-1 interaction with S100P promoted EMT through S100A4 upregulation which was mediated by AKT (33, 91, 92). With the administration of butyrate, organoids demonstrated that the extracellular matrix-integrin/PI3K-Akt axis was involved in CRC cell morphology variation and apoptosis (36).

Butyrate prevented migration and invasion of CRC cells, it was essentially due to the inhibition of HDAC3, which blocked the activation of AKT1 and ERK1/2 (29). Additionally, butyrate interacted with LHX1 to prevent HDAC8 which was up-regulated in tumor tissues. Opposite effects of butyrate on LHX1 mRNA expression occurred in HT-29 and HCT-116 cells despite inhibiting the proliferation of both cell lines (30, 31). Butyrate silenced SIRT-1 belonging to the HDAC family to deactivate mTOR/S6K1 signaling, thus attenuating growth and promoting apoptosis of HCT-116 cells (32). The binding site of IL-6 called gp130 was decreased and occupied by TRAFs which were upregulated by butyrate, leading to inhibition of the JAK2/STAT3 pathway beneficial for CRC (37). Distinguished from other SCFAs, butyrate reversed the excessive expression of prostaglandin EP4 receptors and the production of cyclooxygenase-2 to reduce phenotypic alteration from normal cells to cancer (43). Moreover, butyrate induced CRC cells autophagy through activation of the LKB1-AMPK/ACC signaling pathway and degradation of β-catenin (23, 34).

Attended to altering the epigenetics and metabolic spectrum of CRC cells, butyrate exerted its anti-cancer properties. By regulating the DNA methylation of KEAP1, butyrate blocked NRF2-ARE signaling to enhance its anticancer potential. The change in mitochondrial metabolism and related metabolites participated in the modulation of epigenetics by butyrate (27). Butyrate activated tricarboxylic acid cycle relevant enzymes IDH1 and PDH, thereby the level of downstream product α-KG increased. Considering as a signaling molecule, α-KG affected the demethylation of MSH2 and MLH1 related to apoptosis (93). Meanwhile, α-KG attenuated methylation of DNA and histone H3K4me3, resulting in the Wnt signaling pathway being suppressed in CRC (94). Research showed that enhanced glycolysis and reduced utilization of butyrate in CRC resulted from the decrease of pyruvate kinase M1 (95).

Warburg effect referred to the metabolic adaptation that increased glycolysis in cancer cells and could be obstructed by butyrate-involved metabolic modulation. The membrane content of GLUT1 and cytoplasmic level of G6PD were decreased in response to butyrate and contributed to decreased glucose absorption and utilization, and this process was dictated by the GPR109a-AKT pathway (39). Butyrate-induced inhibition of aerobic glycolysis *via* promoting tetramerization and dephosphorylation of PKM2, thus reversing metabolic dominance in cancer cells (28). For CRC cells, the metabolic change in response to butyrate was reflected in promoting oxidative metabolism rather than glycolysis (38). In addition, iron death is the way iron-dependent cells die programmatically, and butyrate induces iron death in CRC cells through the CD44/SLC7A11 signaling pathway (96).

### 3.4 Hepatocellular carcinoma

As a significant hazardous factor, Hepatitis B Virus (HBV) fosters the progression of hepatocellular carcinoma (HCC). Butyrate substantially suppressed the proliferation of Hep G2.2.15 cells and replication of resident HBV by inhibiting SIRT-1 and thereby promoting p53 acetylation (40). HBx, an oncogenic protein encoded by HBV, may lead to the accelerated occurrence and development of HCC in multiple ways. Along with the downregulation of HBx-related pathways, SCFAs resulted in the incremental expression of tumor suppressor DAB2, which suppressed RAS activity and thus delayed the progress of HCC (41). Previous studies indicated that butyrate inhibited the AKT/mTOR pathway by increasing ROS generation, thus contributing to apoptosis and autophagy of Huh 7 cells (97). The HCC mice intervened with fecal *Lactobacillus reuteri* transplantation appeared to postpone cancer progression. The related mechanism was that acetate produced by *Lactobacillus reuteri* metabolism inhibited the production of IL-17A, the effector molecule of group 3 innate lymphoid cells, through HDAC inhibition and induction of Sox acetylation. Separately, in combination with SCFAs, PD-1 inhibitors showed an enhanced antitumor effect in HCC mice (14). *Lachnospiracea* had the effect of reducing liver fibrosis, which was partly due to SCFAs mediated. It has been proved that oral SCFAs can inhibit fibrosis in mdr^2−/−^ mice treated with vancomycin (98). Moreover, propionate may enhance the chemotherapeutic efficacy of conventional chemotherapeutic agents in HCC. Studies showed that propionate induced TNF-α expression by activating GPR41 and increased cisplatin-induced activation of caspase-3, thereby mediating HepG2 apoptosis (99). The 16s RNA expression of butyrate-producing bacteria in HCC patients decreased, and butyrate supplementation could promote apoptosis and inhibit proliferation in HepG2 cells. More than that, butyrate may enhance the therapeutic potential of sorafenib which has shown therapeutic efficacy by targeting miR-7641 and miR-199, whose expression could be reduced with butyrate treatment (42). By reducing HK2 dependent on c-myc signaling, butyrate resisted glycolysis to enhance the efficacy of sorafenib (44). For HCC patients treated with lenvatinib, the metabolism of butyrate in patients without diarrhea and other adverse reactions was relatively rich and active (100). The latest research indicated that acetate supplementation could induce the level of NAT2 in HepG2 cells, similar to glucose and insulin which led to changes in metabolism-related genes (101). Not only that, butyrate showed the same anti-cancer effect in cholangiocarcinoma cells. Butyrate and HDAC6 inhibitors have synergistic effects on preventing proliferation, migration, and EMT (45).

Alcoholic fatty liver disease (AFLD) and nonalcoholic fatty liver disease (NAFLD) can progress to cirrhosis, eventually turning into liver cancer. Butyrate inhibited gasdermin D-mediated pyroptosis to improve intestinal barrier disruption and endotoxemia, thereby attenuating hepatic steatosis and inflammation in AFLD (102). Moreover, butyrate induced alteration of the hepatic lipid profile and alleviated hepatic steatosis to treat NAFLD relying on the regulation of the LKB1-AMPK-Insig signaling pathway (103). Taken together, SCFAs restricted the evolution of HCC in the premalignant stages.

### 3.5 Pancreatic cancer

It is found that the levels of propionate and butyrate were reduced and the composition of fecal microbiota was altered in patients with pancreatic ductal adenocarcinoma compared to controls (104, 105). Targeting tumor-specific immune cells, SCFAs showed strong anti-cancer effects either in isolation or in combination with other tumor remedies. It was shown that associated with HDAC inhibition, butyrate and pentanoate upregulated the production of IL-2, CD25, and mTOR, which were involved in the regulation of T cell activation. With triggering augmented effector molecules, butyrate and pentanoate also increased the tumor-killing capacity of CTLs. In addition, SCFA also shows excellent prospects in adoptive immune therapy. ROR1-CAR T cells pretreated with butyrate or pentanoate showed a better therapeutic effect in the pancreatic cancer mouse model (9). Another study showed that butyrate delayed the development of carcinoma by reversing the immunosuppressive function of CD11b cells and enhancing the immune function of CD8+T cells in pancreatic ductal adenocarcinoma patients (46). The influence of SCFAs was not only limited to TIME but also extended to tumor-associated genes. After the treatment of butyrate, the upregulation of p16INK4a, p14ARF, and p15INK4b mediated by inhibiting HDACs activities can be observed in AsPC-1 (47). In BxPC-3 and PANC-1 cell lines, butyrate inhibited proliferation and induced apoptosis, either alone or in combination with gemcitabine. Especially, combined medication alleviated gastrointestinal mucosa, liver, and kidney damage caused by gemcitabine. Because of the inhibition of HDACs, butyrate also modulated TIME-related components (49). SCFAs can regulate multiple signal pathways to interfere with the progress of pancreatic cancer, and may also be used as early cancer predictive markers (106).

## 4 Targeting microbiota-SCFAs axis for treatments of gastrointestinal tumors

Probiotics can fight against gastrointestinal tumors by increasing the abundance of gut microbiota, regulating the activities of some enzymes that contribute to the production of carcinogenic compounds and improving the intestinal barrier (107). 23 randomized controlled trials indicated that diverse probiotics supplementation ameliorated symptoms and improved life quality, as well as reduce the adverse reactions of traditional treatment of patients with CRC (108). Certainly, increasing the production of SCFAs is a pivotal way. Screening potential probiotic *Streptococcus salivarius* from human colostrum can inhibit the proliferation of CRC cells by more than 55%. *Streptococcus salivarius* adhered directly and induced apoptosis in cancer cells, promoted the production of SCFAs, and regulated the activated B and T lymphocytes (109). *C. butyricum* restrained the progression of gastrointestinal tumors relying on butyrate. *C. butyricum* modulated Wnt/β-catenin signaling in Apc^min/+^ mice to reduce high-fat diet-induced CRC, suppressed colitis-associated colon cancer *via* inhibiting the NF-κB pathway, and enhanced the ICIs curative effect on lung cancer (85, 110, 111). *Propionibacterium freudenreichii* induced the intrinsic apoptosis of CRC cells by producing propionate and acetate which act on mitochondria (112). *Pediococcus acidilactici* UAMS, as a high butyrate-producing bacterium, was isolated from bhaati jaanr and inhibited the proliferation of HT29 and SW480 (113). *Roseburia intestinalis*, *Faecalibacterium prausnitzii*, *Lactiplantibacillus plantarum*, and *Eubacterium callanderi* have also been proven to enhance the anti-cancer immune response in CRC accompanied by butyrate production (114–117). Lactic acid-producing bacteria played a major role in controlling intestinal carcinogenesis because of SCFAs synthesis (118). Treatment with VSL#3 probiotics increased the levels of propionate and butyrate, resulting in recruiting Th17 cells *via* the CCL20/CCR6 axis to attenuate lung metastasis of melanoma (119). *Lactobacillus rhamnosus* GG ATCC 53103, *Limosilactobacillus reuteri* DSM 17938, *Lactobacillus johnsonii* LC1 and other probiotics not only inhibited CRC cells proliferation but also improved chemotherapy responsiveness (120). Therefore, supplementation of probiotics can increase SCFAs to a certain extent, and then play an essential role in cancer prevention and management.

As the precursor of SCFAs, dietary fiber is one of the representative prebiotics. Dietary fiber is a kind of nutrient that naturally exists in plants and cannot be digested and absorbed by the human intestinal tract. It can reduce the incidence rate and death risk of cardiovascular disease, cholelithiasis, diabetes, cancer, and other diseases. Research has corroborated that taking more dietary fiber decreased the risk of multiple diseases including CRC. There is a statistically significant and strong correlation between dietary fiber consumption and the chance of developing colorectal adenoma and CRC (121–123). As a representative dietary fiber, pectin significantly increased the diversity of gut microbiota, especially butyrate-producing bacteria, and promoted T cell infiltration in TIME, thus enhancing the anti-programmed death-1 (anti-PD-1) monoclonal antibody (mAb) effect (124). Omega-3 polyunsaturated fatty acids (PUFAs) that cannot be synthesized by the human body and must be ingested from food, have been proven to improve hyperlipidemia, coronary heart disease, and atherosclerosis. The increase of butyrate-producing bacteria abundance could be observed when daily taking more PUFAs. Meanwhile, probiotics *Lactobacillus* increased while *Fusobacterium nucleatum* decreased. Studies have shown that some PUFAs can assist chemotherapeutic drugs 5-FU and oxaliplatin for colorectal cancer and reduce side effects (125, 126).

In addition to supplementing probiotics, prebiotics, and synbiotics, FMT may also be an option to increase SCFAs and resist gastrointestinal tumors. In carcinogen-inducing conventional mice or germ-free mice, FMT from patients with CRC reduced gut microbiota richness and promoted gastrointestinal tumor formation (127). Treatment of dextran sulfate sodium or azoxymethane can induce CRC in laboratory mice, and FMT from wild mice to experimental mice could improve this process (128). Although there was no clinical evidence that FMT can directly treat CRC, a study suggested that FMT can help improve the efficacy of chemotherapy. Transplanting the feces of healthy donor mice into FOLFOX-treated mice restored the composition of fecal intestinal microbiota destroyed after FOLFOX treatment and reduced the severity of diarrhea and intestinal mucosal inflammation (129). Furthermore, FMT confirmed the auxiliary effect of pectin on anti-PD-1 mAb (124). FMT enhanced the anti-PD-1 therapy efficacy by increasing the diversity of microbiota and modulating immune function (130). FMT is the most direct way to shape the microbiome, the direct increase of SCFAs-producing bacteria through FMT provides a broad prospect for the treatment of gastrointestinal cancer.

The concentrations of SCFAs and the abundance of SCFAs-producing bacteria in patients who responded to anti-PD-1 immunotherapy were significantly higher than those in non-responders (131). Most recently, a cohort study has confirmed that SCFAs assumed potential biomarkers for identifying solid tumor patients who may benefit from PD-1 inhibitors (Nivolumab or Pembrolizumab) treatment (132). Although ICIs have been demonstrated in clinical practice with great success, it is accompanied by a wide range of adverse reactions, among which cardiotoxicity is the deadliest. Supplementation of butyrate and recolonization of *Prevotella loescheii* relieved the cardiotoxicity and gut microbiota dysbiosis induced by BMS-1 (133). Nevertheless, the extent to which SCFAs affect ICIs has remained controversial. A high concentration of blood butyrate and propionate reduced the anti-cytotoxic T lymphocyte-associated antigen-4 (CTLA-4) efficacy whether in mice models or in patients. By restraining dendritic cell maturation and T cell accumulation in TIME, SCFAs limited the therapeutic effect of anti-CTLA-4 mAb (**Figure 2**) (134).

In addition to immunotherapy, SCFAs also modulate tumor responsiveness to radiochemotherapy and immunotherapy. Clinical research found that after preoperative neoadjuvant radiochemotherapy, responder CRC patients show more enriched levels of butyrate-producing bacteria and SCFAs in the feces than those who did not (135). Butyrate can be used as the synergist of oxaliplatin to synergistically enhance the anti-cancer effect (136). Butyrate directly facilitated the chemotherapy efficacy of oxaliplatin by regulating CD8^+^ T cells. In addition, the level of butyrate was high in the serum of responder patients compared to non-responder patients in CRC (61). Compared with radiation administration alone, the radiation-butyrate combination significantly enhanced the anti-cancer effect. Butyrate could induce cell cycle arrest by promoting FOXO3A-mediated transcription, alongside protecting normal cells from radiation damage. Since HDACs inhibitors have been proven to enhance the sensitivity of radiotherapy, it may be speculated that butyrate enhanced the efficacy of radiotherapy because of the inhibition of HDACs. Butyrate also enhanced the therapeutic effect of 5-FU on CRC by suppressing Warburg Effect, this process was due to the activation of GPCRs (137). Butyrate could also enhance the efficacy of 5-FU through the GPR109a-AKT signal pathway (39). Meanwhile, SCFAs impaired the pro-inflammatory effect of 5-FU and increased the expression of TJ protein in the mucosa (138). However, abnormal activity of butyrate-producing bacteria and excessive butyrate in patients induced resistance to chemotherapeutics. It is demonstrated that CRC cell lines that were resistant to butyrate showed obvious chemoresistance (139). More clinical trials are being explored (**Table 2**).

**Table 2:** Approved clinical trials explore the effects of short-chain fatty acids on the treatment and prognosis of patients with colorectal cancer (Data sources: ClinicalTrials).

Identifier	Launch date	Conditions	Gender	Age	Study Title	Interventions	Treatment duration phase	Study design	Measures	Outcomes
NCT04211766	2021.01	Healthy subject	M/F	50 years to 75 years	Fiber and fish oil supplements for the prevention of colorectal cancer	Dietary supplement: dietary fiber; Dietary supplement: fish oil Other: placebo;	Phase 1	Allocation: randomized; Intervention model: crossover assignment; Masking: double (participant, investigator); Primary purpose: prevention;	Change in mRNA expression profiles in the exfoliome at baseline to 2 years	Completed No results posted
NCT03781778	2019.05	Colon cancer; Rectal cancer; Survivor;	M/F	18 years and older	Pilot trial of resistant starch in stage I-III olorectal cancer survivors	Other: dietary intervention (resistant starch); Other: dietary intervention (regular starch); Other: questionnaire administration;	Phase 2	Allocation: randomized; Intervention model: parallel assignment; Masking: double (participant, investigator); Primary purpose: prevention;	Variability of biomarkers of insulin resistance and inflammation (adiponectin) at baseline and 8 weeks; Variability in biomarkers of insulin resistance and inflammation (CRP) at baseline and 8 weeks; Variability in gut microbial communities from human stool samples at 2 and 8 weeks; Fecal microbiota from resistant starch responders at 2 and 8 week;s	Completed The variation of adiponectin and CRP and the diversity of gut microbiota are low in resistant starch group
NCT03028831	2017.12	Colon cancer	M/F	40 years to 70 years	Fiber to reduce colon cancer in Alaska native people	Dietary supplement: 70g of fully digestible starch amylopectin corn starch; Dietary supplement: resistant starch;	Not applicable	Allocation: randomized; Intervention model: parallel assignment; Masking: quadruple (participant, care provider, investigator, outcomes assessor); Primary purpose: prevention;	Colonic mucosal proliferation at 4 weeks; Colonic microbiota at 4 weeks; Colonic secondary bile acids at 4 weeks;	Recruiting
NCT03531606	2016.12	Sigmoid colon cancer	M/F	20 years to 75 years	The effects of Mechnikov probiotics on symptom and surgical outcome	Drug: mechnicov probiotics; Drug: placebo;	Not applicable	Allocation: randomized; Intervention model: parallel assignment; Masking: single (care provider); Primary purpose: treatment;	Anterior resection syndrome improvement change at 1, 4, 5 weeks after surgery; Bowel examination at 4, 5 weeks after surgery; Quality of life of cancer patients (EORTC QLQ-C30) at 1 week before surgery and 4 weeks after surgery; Markers related inflammation at 1 week before surgery and 4 weeks after surgery; NSI at 1 week before surgery and 1.5, 4 weeks after surgery; Clavien-Dindo classification at 1 week before surgery and 1.5, 4 weeks after surgery; NGS, short chain fatty acids at 1 week before surgery and 1, 4, 5 weeks after surgery;	Completed No results posted
NCT02699047	2015.03	Gastrointestinal cancer; Colorectal cancer; Stomach cancer;	M/F	40 years to 79 years	Fish oil supplementation in gastrointestinal cancer	Dietary supplement: encapsuled fish oil; Dietary supplement: encapsulated olive oil;	Not applicable	Allocation: randomized; Intervention model: parallel assignment; Masking: quadruple (participant, care provider, investigator, outcomes assessor); Primary purpose: treatment;	Change in quality of life at baseline and 9 weeks (final moment); Cytokines of inflammatory response at baseline, 5 and 9 weeks; Body weight at 5 and 9 weeks; Body mass index at baseline, 5 and 9 weeks; Weight change at baseline, 5 and 9 weeks; Fat mass at baseline and 9 weeks; Lean body mass at baseline and 9 weeks arm circumference at baseline, 5 and 9 weeks; Tricipital skinfold at baseline, 5 and 9 weeks; Serum C-reactive protein at baseline, 5 and 9 weeks; Activity of catalase at baseline and 9 weeks; Activity of glutathione peroxidase at baseline and 9 weeks; Activity of superoxide dismutase at baseline and 9 weeks; Lipid peroxidation at baseline and 9 weeks; Evaluation of adverse events consequences at baseline and 9 weeks; Graduation of adverse events at baseline, 5 and 9 weeks; Tumor markers at baseline and 9 weeks; Survival at baseline, 6 months and one year; Serum albumin at baseline, 5 and 9 weeks;	Unknown
NCT01661764	2013.02	Colorectal adenomatous polyps	M/F	40 years to 79 years	Fish oil supplementation, nutrigenomics and colorectal cancer prevention	Drug: eicosapentanoic acid and docosahexanoic acid; Drug: oleic acid; Other: placebo;	Phase 2	Allocation: randomized; Intervention model: factorial assignment; Masking: quadruple (participant, care provider, investigator, outcomes assessor); Primary purpose: prevention;	Rectal epithelial ecll proliferation at 6 month; Rectal epithelial cell apoptosis at 6 months;	Completed The results have shown no statistical significance
NCT01575340	2011.07	Colorectal cancer	M/F	19 years and older	Study to assess the effect of consumption of fish oil Encapsulated on inflammatory markers in colorectal cancer	Dietary supplement: fish oil encapsuled	Not applicable	Allocation: randomized; Intervention model: parallel assignment; Masking: none (open label); Primary purpose: supportive care;	Change in inflammatory markers at two months; Change in body composition at two months; Change in nutritional status at two months; Changes in cellular lipid profile at two months;	Completed No results posted
NCT01479907	2010.11	Colorectal neoplasms	M/F	18 years to 80 years	Synbiotics and gastrointestinal function related quality of life after colectomy for cancer	Dietary supplement: synbiotics; Dietary supplement: placebo;	Not applicable	Allocation: randomized; Intervention model: single group assignment; Masking: quadruple (participant, care provider, investigator, outcomes assessor); Primary purpose: supportive care;	Assessment of gastrointestinal function-related quality of life at 1, 3 and 6 months postoperatively	Completed No results posted
NCT03072641	2010.06	Colon cancer	M/F	18 years and older	Using probiotics to reactivate tumor suppressor genes in colon cancer	Dietary supplement: ProBion Clinica	Not applicable	Allocation: randomized; Intervention model: parallel assignment; Masking: none (open label); Primary purpose: basic science;	Changes in microbiota composition at baseline and after probiotics use; Epigenetic changes at baseline and after probiotics use;	Completed No results posted
NCT01609660	2010.03	Colorectal cancer	M/F	18 years and older	Impact of probiotics on the intestinal microbiota	Dietary supplement: *Saccharomyces boulardii*	Phase 4	Allocation: randomized; Intervention model: parallel assignment; Masking: none (open label); Primary purpose: prevention;	Mucosal cytokine; Short chain fatty acids;	Completed No results posted
NCT03420443	2008.11	Rectal cancer	M/F	18 years to 80 years	Action of synbiotics on irradiated GI mucosa in rectal cancer treatment	Dietary supplement: oat bran; Dietary supplement: oat bran and blueberry husks; Dietary supplement: no oral supplementation;	Not applicable	Allocation: randomized; Intervention model: parallel assignment; Masking: triple (participant, investigator, outcomes assessor); Primary purpose: prevention;	Action of synbiotics on irradiated gastrointestinal mucosa in rectal cancer treatment at 2 weeks	Completed No results posted
NCT00335504	2006.03	Colon cancer Rectal cancer	M/F	40 years and older	Atorvastatin calcium, oligofructose-enriched inulin, or sulindac in preventing cancer in patients at increased risk of developing colorectal neoplasia	Drug: oligofructose-enriched inulin; Drug: sulindac; Drug: placebo; Drug: atorvastatin calcium; Other: laboratory biomarker analysis;	Phase 2	Allocation: randomized; Intervention model: parallel assignment; Masking: double (participant, investigator); Primary purpose: prevention;	Percent change in number of rectal aberrant cryptic foci as measured by magnification chromoendoscopy at 6 months; Effects on proliferation (Ki67 expression) at 6 months; Effects on apoptosis (caspase-3 expression) at 6 months; Adverse events at 6 months;	Completed The results have shown no statistical significance

M, male; F, female; AJCC, American Joint Committee on Cancer; CRP, C-reactive protein; EORTC QLQ-C30, The European Organisation for Research and Treatment of Cancer core questionnaire-C30; NSI, Nutritional Screening Index; NGS, next-generation sequencing.

## 5 Conclusions and perspectives

Immune cells and molecules have become highlights of tumor treatment. ICIs, established in TIME, targeted kill tumor cells by regulating autoimmune cells and immune molecules while reducing damage to normal tissues, and have become the representative of tumor immunotherapy. Gut microbiota and its metabolites affect the sensitivity and responsiveness of the host to anti-cancer therapy. SCFAs serve as decisive products of gut microbiota and have been proven to inhibit the proliferation, invasion, and migration and induce apoptosis of gastrointestinal tumor cells. The latest findings support the potential of SCFAs in immunotherapy. SCFAs can modify the differentiation and function of immune cells as well as the production and release of cytokines, and control tumor growth and metastasis by multiple signal pathways. In addition, SCFAs can also help to improve the therapeutic effect of radiotherapy and chemotherapy and reduce adverse reactions. However, clinical trials are relatively insufficient at present, and the polymicrobial combination leads to large individual differences in therapeutic effects. The supplement of specific foods and probiotics can assist complex anti-tumor treatment, enhance the curative effect and improve the prognosis. FMT is the most direct transformation method of microbial composition, which may become a new part of complex tumor treatment in the future.

## Author contributions

YD collected literature, drafted the manuscript, and prepared figures. KXZ modified figures and edited the manuscript. JGW, XW, and YYD collected data, and designed and made tables. HQH and JYW provided critical feedback and helped shape the manuscript. TYL, BMW, and HLC reviewed the manuscript and provided funding acquisition. All authors contributed to the article and approved the submitted version.

## Glossary

**Table:**

AcK	acetate thiokinase
ACC	1-aminocyclopropane-1-carboxylic acid
ACSS2	acetyl-CoA synthetase 2
AhR	aryl hydrocarbon receptor
α-KG	α-ketoglutarate
AFLD	alcoholic fatty liver disease
AKT	the protein kinase B
AMPK	AMP-activated protein kinase
ARE	antioxidant response element
BIRC5	baculoviral inhibitor of apoptosis repeat-containing 5
BMI-1	B cell-specific MLV integration site-1
CAR	chimeric antigen receptor
CRC	colorectal cancer
CSE1L	chromosome segregation 1-like
CTLA-4	cytotoxic T lymphocyte-associated antigen-4
CTLs	cytotoxic T lymphocytes
DAB2	disabled homolog 2
EHMT2	euchromatic histone-lysine N-methyltransferase 2
EIF4G2	eukaryotic translation initiation factor 4 gamma 2
EMT	epithelial-to-mesenchymal transition
EP4	E-prostanoid receptor 4
ERK1/2	extracellular signal-regulated kinase 1/2
FFAR	fatty acid receptor
FMT	fecal microbiota transplantation
FOXP3	Forkhead box P3
5-FU	5-Fluorouracil
G6PD	glucose-6-phosphate dehydrogenase
GLUT1	glucose transporter type 1
gp130	glycoprotein 130
GPCRs	G protein-coupled receptors
GSK-3β	glycogen synthase kinase-3β
HBV	hepatitis B virus
HBx	hepatitis B x antigen
HCC	hepatocellular carcinoma
HCP1	heme carrier protein 1
HDACs	histone deacetylases
HECTD2	HECT domain E3 ubiquitin protein ligase 2
HIF	hypoxia inducible factor
HRE	hypoxia-response element
ICIs	immune checkpoint inhibitors
ID2	inhibitor of DNA binding 2
IDH1	isocitrate dehydrogenase 1
IFN-γ	interferon-γ
IL	interleukin
ITIM	immunoreceptor tyrosine-based inhibition motif
JAK2	janus kinase-2
KEAP1	Kelch-like ECH-associated protein 1
LHX1	LIM homeobox 1
LKB1	liver kinase B1
mAb	monoclonal antibody
MCT1	monocarboxylic transporter 1
MICA/B	major histocompatibility complex-class I chain related proteins A and B
miRNA	microRNA
MLH1	mutL homolog 1
mTOR	mammalian target of rapamycin
MSH2	MutS homolog 2
NAFLD	nonalcoholic
	
NKG2D	natural killer group 2, member D
NRF2	nuclear Factor erythroid 2-related factor 2
PD-1	programmed death-1
PDH	pyruvate dehydrogenase
PDK	phosphoinositide-dependent kinases
PEPCK-M	mitochondrial phosphoenolpyruvate carboxykinase
Per	period circadian regulator
PI3K	phosphoinositide 3-kinase
PKM2	pyruvate kinase isoform 2
PRMT1	protein arginine methyltransferase 1
PUFAs	Omega-3 polyunsaturated fatty acids
PVR	poliovirus receptor
ROR1	the receptor tyrosine kinase-like orphan receptor 1
RORγt	retinoid-related orphan receptor-γt
ROS	reactive oxygen species
S6K	ribosomal S6 kinase
SCFAs	short-chain fatty acids
SIRT-1	Sirtuin 1
SMCT-1	sodium-coupled monocarboxylate transporter 1
STAT3	signal transducer and activator of transcription 3
TGF-β	transforming growth factor-β
Th	T helper
TIME	tumor immune microenvironment
TNF-α	tumor necrosis factor-α
TNFAIP1	Tumor necrosis factor alpha-induced protein 1
TRAFs	tumor necrosis factor receptor-associated factors
Treg	T regulatory
Trx-1	thioredoxin-1
